# Low Nephron Number and Its Clinical Consequences

**DOI:** 10.5041/RMMJ.10061

**Published:** 2011-10-31

**Authors:** Valerie A. Luyckx, Khuloud Shukha, Barry M. Brenner

**Affiliations:** 1* Associate Professor, Division of Nephrology, University of Alberta, Edmonton, Alberta, Canada;; 2 Internal Medicine Resident, Mount Auburn Hospital, Cambridge, MA, USA; and; 3 Samuel A. Levine Distinguished Professor of Medicine Renal Division, Brigham and Women’s Hospital, Harvard Medical School, Boston, MA, USA

**Keywords:** Low birth weight, nephron number, developmental programming, hypertension, renal disease, kidney size

## Abstract

Epidemiologic studies now strongly support the hypothesis, proposed over two decades ago, that developmental programming of the kidney impacts an individual’s risk for hypertension and renal disease in later life. Low birth weight is the strongest current clinical surrogate marker for an adverse intrauterine environment and, based on animal and human studies, is associated with a low nephron number. Other clinical correlates of low nephron number include female gender, short adult stature, small kidney size, and prematurity. Low nephron number in Caucasian and Australian Aboriginal subjects has been shown to be associated with higher blood pressures, and, conversely, hypertension is less prevalent in individuals with higher nephron numbers. In addition to nephron number, other programmed factors associated with the increased risk of hypertension include salt sensitivity, altered expression of renal sodium transporters, altered vascular reactivity, and sympathetic nervous system overactivity. Glomerular volume is universally found to vary inversely with nephron number, suggesting a degree of compensatory hypertrophy and hyperfunction in the setting of a low nephron number. This adaptation may become overwhelmed in the setting of superimposed renal insults, e.g. diabetes mellitus or rapid catch-up growth, leading to the vicious cycle of on-going hyperfiltration, proteinuria, nephron loss and progressive renal functional decline. Many millions of babies are born with low birth weight every year, and hypertension and renal disease prevalences are increasing around the globe. At present, little can be done clinically to augment nephron number; therefore adequate prenatal care and careful postnatal nutrition are crucial to optimize an individual’s nephron number during development and potentially to stem the tide of the growing cardiovascular and renal disease epidemics worldwide.

In 1988, Brenner, Anderson, and Garcia suggested that a low nephron number, acquired *in utero*, may be a common denominator in populations with high susceptibility to hypertension and renal disease.^1^ Such a kidney with fewer nephrons, and hence a low filtration surface area, would have a reduced capacity to excrete sodium, inducing a hypervolemic state, thereby contributing to the development of hypertension (Figure 1). Animal experiments and epidemiological data have accumulated in support of this “nephron number” hypothesis.^2^–^8^ Nephron number varies surprisingly widely among individuals, more, for example, than height or weight, with a variability of up to 10-fold within select populations.^5^,^6^,^9^–^17^ An individual’s nephron number is the result of a complex interplay between genetics and environment that plays out through their lifetime, carrying the imprint of their past, being reflected in their present renal function, and impacting their future risk of hypertension and kidney disease.

**Figure 1:**
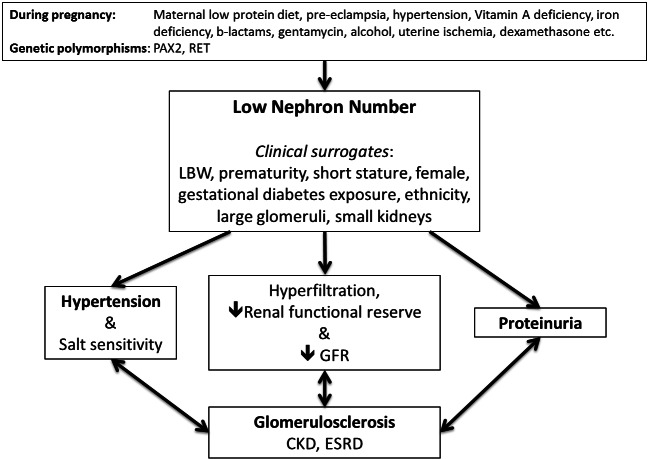
**Known causes of low nephron number.** Schematic diagram outlining the known causes of low nephron number, derived from human and animal studies, current clinical surrogate markers for low nephron number, and clinical consequences of low nephron number, reduced filtration surface area, and abnormal glomerular development.

## DETERMINANTS OF NEPHRON NUMBER

### Prenatal Life and Birth Weight

Kidney development in humans begins in the 9th week and ends around the 36th week of gestation.^5^ There is no evidence for postnatal nephrogenesis in humans, except in extremely preterm infants in whom abnormal nephrogenesis was observed until day 40 after birth.^16^,^18^ Similarly, in preterm baboons followed for 21 days after birth, nephrogenesis did continue, but the proportion of immature, poorly vascularized and abnormal glomeruli was increased compared to gestational controls.^19^ In young adult rats exposed to a low protein diet *in utero*, glomerulogenesis was retarded with a higher proportion of immature nephrons, associated with abnormalities in the glomerular basement membrane and podocyte structure.^20^ These authors postulate that such subtle structural abnormalities programmed early on may increase susceptibility to on-going renal injury.

Numerous studies have investigated events and changes during pregnancy that lead to reduced nephron number, including maternal diets deficient in protein, iron, or vitamin A, uterine artery ligation, maternal hyperglycemia, prenatal exposure to glucocorticoids and drugs such as gentamycin, cyclosporin, β-lactams, ethanol, and cox2 inhibitors.^21^–^36^ Many of these interventions also result in low birth weight (LBW) offspring.

The World Health Organization defines LBW as a birth weight under 2,500 g; thus an infant can have a LBW by being born premature (before the 37th week of gestation), although at an appropriate weight for gestational age (AGA), or due to intrauterine growth restriction (IUGR) during a term pregnancy.^37^ A small for gestational age infant (SGA) is defined as weighing less than the 10th percentile of the normal weight for gestation.^37^ Risk factors for LBW are many: in the Third World mostly related to maternal malnutrition, inadequate prenatal care, infections, etc., and in the First World also related to higher-risk pregnancies, prematurity, and advanced maternal age.^37^–^40^ Interestingly, maternal LBW in both whites and blacks in the US was a risk factor for infant LBW, prematurity, and IUGR, regardless of economic environment, demonstrating the impact of developmental programming across generations.^41^

In humans, nephron numbers were found to be lower in neonates with LBW.^12^,^16^,^42^ Gestational age also correlates with nephron number, and prematurity results in reduced nephron endowment.^16^ In adults, nephron number has not been reported in those of LBW, but several studies have shown a strong direct correlation with birth weight across the normal birth weight range among Australian Aborigines, Caucasians, and people of African origin.^11^,^12^,^16^,^43^ One large study calculated an increase of 257,426 glomeruli per kilogram increase in birth weight.^11^ More human studies are required including diverse populations and a broad spectrum of birth weights to define further this relationship.

At the other extreme, high birth weight (HBW), defined as a birth weight > 4,000 g, has also been associated with adverse long-term renal outcomes, although the relationship with nephron number in humans is not known.^44^,^45^ HBW is often the result of maternal hyperglycemia, and, in animals, offspring of diabetic dams have been found to have reduced nephron numbers.^46^–^48^

### Genetics

Important pathways in nephrogenesis include GDNF/RET, FGF, PAX2, HH, and others which have been expertly reviewed elsewhere.^49^ Polymorphisms in several of these genes have been investigated in relation to kidney size and nephron number in humans. PAX2 has a wide range of functions in kidney development, and a common variant in the population, the AAA haplotype, reduces PAX2 mRNA expression and causes a 10% reduction in kidney volume.^50^,^51^ Similarly, RET is essential for branching nephrogenesis, and a polymorphic variant, *RET*^1476A^, is associated with an almost 10% reduction in kidney volume at birth.^52^ Kidney volume was found to be proportional to nephron number in this study. Mutations in other genes such as Imx-1, Eya-1, Six1, Sall1, and tcf2 also result in a reduced nephron number, small kidney size, and disorganized renal tissue, in addition to other extrarenal manifestations.^53^

## OTHER CLINICAL CORRELATES OF NEPHRON NUMBER

Except for rough estimates of the nephron number by renal MRI or kidney biopsy, an accurate count can only be done post mortem.^15^,^43^,^54^ In humans, thus far, LBW and prematurity are the strongest clinical correlates of low nephron number. In animals, however, low nephron number has been reported in the absence of LBW, and, conversely, not all LBW animals have reduced nephron numbers; therefore birth weight alone may not be a universal surrogate marker for nephron number.^55^,^56^ Several additional clinical surrogates for nephron number have been examined (Figure 1), which, although not absolute, may also serve to increase awareness of the possibility of low nephron number, with the attendant risks for hypertension and renal disease, and may therefore impact on optimization of other risk factors.

### Anthropomorphic Factors

Females are estimated to have 12% fewer glomeruli than males.^43^,^57^,^58^ Increasing age is associated with a predicted reduction of 3,676 glomeruli per kidney per year after age 18.^15^,^43^ Adult height has been found to correlate positively with nephron number, with an estimated increase of 28,000 glomeruli per centimeter increase in height, and height was found to contribute to two-thirds of the variance in glomerular number.^15^,^43^,^58^ Birth weight tends to be associated with subsequent height and therefore may be a confounder in this relationship, but adult height is much more readily available than birth weight in clinical practice and therefore useful.^59^

### Glomerular Volume

Although nephron number does not increase after birth, the kidney matches its filtration capacity to the body’s demands by increasing the glomerular size through hypertrophy.^5^,^11^,^12^,^57^,^58^ As such, mean glomerular volumes have consistently been found to correlate *inversely* with glomerular number and *directly* with current body size.^43^ Keller et al. reported a 133% higher mean glomerular volume and a 46.6% reduction in glomerular number in hypertensive subjects, compared to controls.^6^ Lower nephron number, black race, hypertension, and body size all correlate with an increase in individual glomerular volume within a single kidney.^14^,^17^,^43^ Larger glomeruli therefore suggest a lower nephron number, although in African-Americans glomerular volume appears to be globally increased but still in inverse proportion to nephron number, suggesting additional factors contribute to glomerular size in this population.^9^

### Kidney Mass

Kidney weight can only be measured *ex vivo*, but from autopsy studies, nephron number correlates directly with kidney weight in adults and children.^15^,^52^ Zhang et al. predicted an increase of 23,459 glomeruli per gram of kidney mass (95% confidence interval 4,590–42,238) in infants 3 months and younger.^52^ In living subjects, kidney mass is obtainable by weighing the donor kidney prior to transplantation and has proven to have clinical relevance (*vide infra*).

### Kidney Volume

Kidney volume can be readily measured by ultrasound. Spencer et al. found relatively lower kidney volumes in LBW Australian Aboriginal children aged 5–18 when adjusted for body size.^60^ In contrast, Rakow et al. did not find a significant difference between kidney volumes of individuals who had been term AGA, term SGA, or preterm, when adjusting for body surface area (BSA), gender, and age.^61^ Kidney size is dependent on nephron number and the degree of nephron hypertrophy and is strongly correlated with current body size.^15^ In fetuses and at birth, kidney volume is proportional to nephron number; however, subsequently, normal kidney growth (impacted by BSA, age, and gender), glomerulomegaly (hypertrophy due to low nephron number, obesity, etc.), and nephron loss through injury are likely to affect kidney volume disproportionately, making a direct relationship less clear.^60^ Among young adults born prematurely (either AGA or SGA) compared with term age-matched controls, prematurity was associated with smaller kidneys at age 20 years, whereas IUGR had only a small, non-significant effect.^62^ Kidney volume may therefore not be ideally reliable as a surrogate for nephron endowment.

## CLINICAL CONSEQUENCES OF IMPAIRED DEVELOPMENTAL PROGRAMMING IN THE KIDNEY

### Nephron Number, Size, and Blood Pressure

In adult animals, surgical removal of one kidney under varying circumstances and in different species does not always result in spontaneous hypertension and renal disease.^63^ In contrast, however, uni-nephrectomy on postnatal day 1 in rats, or fetal uni-nephrectomy in sheep, i.e. loss of nephrons at a time when nephrogenesis is still on-going, does lead to adult hypertension prior to any evidence of renal injury.^64^–^66^ These data support the possibility that intrauterine or congenital reduction in nephron number may elicit different compensatory responses compared to later nephron loss, augmenting the risk of hypertension. Consistent with this view, kidneys from rats that underwent uni-nephrectomy at day 3 of age had similar total number but a greater proportion of immature glomeruli in adulthood, compared with those who underwent nephrectomy at day 120 of age.^67^ In addition, mean glomerular volume in neonatally nephrectomized rats was increased by 59% compared with 20% in adult nephrectomized rats, suggesting a greater degree of compensatory hypertrophy and hyperfunction in response to neonatal nephrectomy. One could argue that as the demand for filtration capacity highly depends on BSA and basal metabolic rate, the increment in BSA from infancy through childhood and adulthood may impose a much greater strain on a smaller kidney early in life, which may demand different and more robust adaptation than during adulthood, when no growth occurs except for changes in weight.^15^

Some suggest that when kidney growth is interrupted, fewer, yet normal, nephrons develop. Others challenge this, as nephrogenesis is a highly complex and regulated process, and expect some structural and/or functional defects in addition to the reduced nephron number. This question is difficult to address, but in GDNF (glial cell-derived neurotrophic factor) heterozygous mice, a model with low nephron number and in which 20% of animals have unilateral renal agenesis, single kidney nephron numbers were found to be identical in mice born with one or two kidneys.^68^ Although glomerular filtration rates (GFR) were similar, salt and water handling were different, suggesting possible alterations in nephron function in the mice with unilateral renal agenesis. In this model, a reduction in nephron number per se was not associated with elevated blood pressures, but when maintained on a high-salt diet GDNF-deficient mice became significantly hypertensive, and blood pressures were highest in those with fewest nephrons.^68^ This observation could be interpreted to suggest that a deficit in nephron number may in itself not be enough to result in disease but likely enhances susceptibility to a second “hit”, transforming subclinical into overt renal dysfunction.^69^

The association between low nephron number and higher blood pressures has been demonstrated in white adults and in Australian Aborigines but has not been proven among individuals of African origin.^6^,^43^,^58^,^70^ To our knowledge, the relationship has not been studied in other ethnic groups. Conversely, a higher nephron number appears to be protective in the Caucasian and Australian Aboriginal populations.^17^,^43^ Similarly, in some animal models restoration of nephron number has been found to abrogate the development of hypertension, suggesting that nephron number is an important factor in the pathogenesis of hypertension.^71^–^73^

### Birth Weight Predicts Later Life Hypertension

Since the 1980s, when the inverse correlation between LBW and hypertension was reported, numerous studies in humans and animals have supported this observation.^2^,^36^,^74^–^79^ It is important to note that in LBW children, blood pressures tend to be higher than those of normal birth weight children but are not in the hypertensive range, but with time blood pressures increase and LBW individuals become overtly hypertensive with age. Although preterm birth itself is associated with increased blood pressure, LBW for gestational age has been more strongly associated with higher blood pressures at birth and at 18 months of age than LBW of prematurity, suggesting that an adverse intrauterine environment is an important factor.^80^–^82^ Consistent with this, a recent Swedish study of 16,265 twins found a correlation between LBW and later life hypertension within dizygotic and monozygotic twin pairs, suggesting that individual fetal growth is an important factor, independent of genetic background, impacting developmental programming of adult disease.^83^ Not all studies confirm the association between birth weight and subsequent blood pressures, however.^84^–^86^ The relationship appears to be least consistent among US black children but is maintained in African and Caribbean black children, suggesting that genetic and/or environmental factors may be more pivotal in the US population.^75^,^77^,^78^,^87^

Importantly, reduced nephron number is not the only link between LBW and hypertension.^3^,^88^ Salt sensitivity has also been shown to be associated with LBW in humans and in some animal models.^68^,^89^,^90^ Altered expression of renal sodium transporters and modulation of the renin–angiotensin–aldosterone system have been shown in prenatally programmed animals, which may contribute to salt sensitivity.^91^–^95^ Consistent with this, in elegant studies Dagan et al. have shown increased tubule sodium transport to be a likely contributor to high blood pressure in adult animals that were exposed to maternal low-protein diet or prenatal dexamethasone.^96^,^97^ Additional proposed mechanisms for developmental programming of blood pressure, studied mostly in animals but also in humans, include increased renal vascular reactivity, altered vascular reactivity, and increased sympathetic nervous system activity.^33^,^98^–^102^

### Birth Weight and Renal Outcomes

#### Proteinuria

Studies in various populations have shown increased urine protein excretion in subjects who had been of LBW, although the significance does not always persist when adjusted for additional risk factors, e.g. current HbA1c in diabetic youth.^80^,^103^ Among Australian Aborigines, albuminuria was found to correlate strongly with LBW and to increase dramatically with age.^104^,^105^ In this population, overt proteinuria was a significant predictor of loss of GFR, renal failure, and natural death.^106^,^107^ Among Pima Indians, a U-shaped association was found between birth weight and albumin excretion in diabetics, i.e. both LBW and HBW (largely due to gestational diabetes) correlated with increased albumin excretion.^44^ Podocyte abnormalities have been described in LBW animals, which may play a role in the development of proteinuria.^20^,^108^ It is likely therefore that intrauterine programming of nephron development may be associated with increased risk of albuminuria.

#### Measures of Renal Function

A reduction in nephron number, in the absence of compensatory hyperfunction, would be expected to result in a lower total GFR and creatinine clearance, and, indeed, in 1-day-old neonates born premature or SGA, GFRs were found to be impaired compared to normal birth weight neonates.^109^ Lower GFR and higher serum creatinine were also found in LBW children, aged 6–12 years, compared with age-matched normal birth weight children.^110^ In contrast, however, no significant difference in GFR was found among three groups of 9–12-year-olds who had been either preterm, term SGA, or term AGA.^61^ Interestingly, in children, GFR measured by cystatin C was found to correlate better with birth weight than creatinine-based formulas, suggesting the validity of these formulas may need to be re-evaluated in LBW individuals.^111^,^112^ A positive correlation was found, however, between birth weight and creatinine-based GFR in a cohort of young adults, born very premature.^80^ Using 24-hour urine creatinine clearance within adult twin pairs, GFRs were found to be lower in the LBW twin, again suggesting an independent effect of the intrauterine environment on programming of renal function.^113^

A small cross-sectional study compared total GFR, effective renal plasma flow, and filtration fraction before and after renal stimulation with low-dose dopamine infusion and oral amino acid intake in 20-year-olds born premature and AGA, premature and SGA, or term and AGA.^114^ It would be expected that a kidney with fewer nephrons is already hyperfiltering to some degree, which may abrogate any change in serum creatinine, but would have a blunted increase in GFR when stimulated further. This study was limited by small sample size, but the relative increase in GFR tended to be lower in SGA compared with AGA and control subjects, and effective renal plasma flow was lower in both SGA and AGA preterm individuals, although not statistically significant.^115^ A recent study of non-diabetic young adults found a significant reduction in renal functional reserve in those with diabetic mothers (i.e. exposed to diabetic milieu *in utero*), compared to those with diabetic fathers, thereby excluding a genetic confounder, and strongly suggesting a long-term impact of gestational diabetes exposure.^114^ The authors postulate that reduced renal functional reserve may reflect a programmed reduction in nephron number in offspring of diabetic mothers. Evaluation of renal functional reserve may therefore be a more sensitive method to detect subtle changes in renal function due to reduced nephron number.

#### Chronic Kidney Disease

A recent meta-analysis of 31 studies found a 70% increase in relative risk of chronic kidney disease (CKD) with LBW.^116^ A U-shaped curve for risk of CKD and birth weight (< 2.5 kg or ≥ 4.5 kg) among adult men, but not women, was found in a large US cohort.^117^ Many animal studies of fetal programming also report increased susceptibility to hypertension and renal dysfunction in males, although the reasons for the gender differences are not entirely clear.^118^ A retrospective study of over 2 million Norwegians reported a relative risk of end-stage renal disease (ESRD) of 1.7 in males and females born below the 10th percentile in weight, but only in females with birth weights > 4.5 kg.^119^ A U-shaped curve was also described between birth weight and ESRD in both males and females in a predominantly black US population.^120^

Epidemiologic studies therefore support the relationship between high or low birth weights and risk of CKD. A relationship between nephron number and risk of CKD in humans, however, has not been directly studied. Hodgin et al. reported renal biopsy findings in six adults who had been born premature and LBW.^121^ They described consistent findings of focal and segmental glomerulosclerosis, associated with glomerulomegaly, most likely on the basis of a congenitally reduced nephron number. Nephron number per se, however, cannot be invoked as the sole cause of renal dysfunction in most patients. A kidney with a reduced nephron complement likely undergoes some degree of hyperfiltration, especially if body size and functional demand are high, and may have subtle structural abnormalities, both of which would enhance susceptibility, or reduce resistance, to additional renal injury or stress (Figure 1). Consistent with this possibility, LBW has been associated with poorer outcomes in patients with nephrotic syndrome, membranous nephropathy, IgA nephropathy, minimal change, and diabetic nephropathy.^45^,^122^–^125^ Abnormal glomerular adaptation and greater renal injury have also been shown in LBW animals with reduced nephron numbers.^108^,^126^ Suggested cellular and molecular mechanisms for the association between LBW and CKD in adult life include an imbalance between apoptosis and cell proliferation, accelerated senescence, and mitochondrial dysfunction.^127^

### Born Small – Stay Small! The Catch-up Effect

The combination of LBW with a rapid increase in weight after birth amplifies the risks for hypertension and cardiovascular disease in later life.^128^–^130^ Rapid weight gain by as early as 2 weeks of age was associated with endothelial dysfunction in the same subjects 16 years later.^131^ The “thrifty phenotype hypothesis” states that in the event of a suboptimal intrauterine environment, embryonic and fetal adaptive responses limit fetal growth, resulting in a phenotype that is better suited to survive under adverse conditions, e.g. nutrient scarcity. These adaptive changes may become maladaptive when the postnatal environment offers better growth conditions, thereby enhancing the risk of hypertension and clinical renal disease.^7^,^132^ Animal models of LBW followed by accelerated postnatal growth have shown enhanced oxidative stress, telomere shortening, and accelerated senescence in kidneys, hearts, and aortas associated with premature death.^133^–^136^ Although more circumstantial, there is evidence pointing to accelerated senescence and increased oxidative stress in LBW humans consistent with “the dangerous road of catch-up growth”.^137^–^140^

### Nephron Dosing in Renal Transplantation

In animal models of renal programming, e.g. maternal gestational low-protein diet or uterine artery ligation, offspring nephron numbers are generally reduced by 25%–30%, often resulting in adult hypertension and renal disease, suggesting that loss of a single kidney (i.e. 50% of nephrons) even in a normal individual, may carry similar risk.^2^,^73^ Indeed, long-term follow-up of 52 kidney donors over 10 years did find an increased risk of hypertension and proteinuria.^141^ Other reports, predominantly in white kidney donors, have reported a lower risk of hypertension, proteinuria, and renal dysfunction, suggesting that uni-nephrectomy is safe.^142^–^145^ More recently, however, warning flags have been raised about the possibility of harm of living kidney donation in other ethnic groups. Among Australian Aboriginal kidney donors, after a median of 16 years, the incidence of hypertension, CKD, and ESRD was very high compared to Caucasian donors.^143^ Similarly, among Aboriginal Canadian donors, the prevalence of hypertension was significantly more frequent than among Caucasians, with 100% of Aborigines having hypertension 20 years after donation.^146^ Estimated GFR was not different between populations in this study, however, although more Aboriginal donors had proteinuria. In US cohorts, hypertension and CKD were significantly more prevalent among black compared to white donors.^147^,^148^ Uni-nephrectomy, therefore, does appear to carry some risk in populations known to be at increased risk of hypertension and kidney disease. These same populations generally have a higher prevalence of extremes of birth weight, low among Australian Aboriginal and US black populations and high in the Canadian Aboriginal population, suggesting that associated low nephron number may be a contributory factor to the increased renal risk post-nephrectomy.

From the recipient’s point of view, the importance of nephron mass as an antigen-independent determinant of transplant outcomes, i.e. matching kidney size to the recipient’s demand, has not always been accepted.^149^ In animal models, independent of immunologic barriers, transplanted nephron mass has a significant impact on allograft survival.^150^–^152^ In humans, various methods have been employed to try to assess the impact of kidney size, utilizing ratios of recipient to donor BSA or body weight, kidney volume to recipient BSA, and kidney weight to recipient weight, on transplant outcomes.^153^–^158^ Several caveats must be borne in mind when interpreting these data: BSA is not always proportional to kidney weight, and two kidneys of the same size may differ in nephron number. The evidence, however, despite the variability in methods, appears to be fairly consistent that small kidneys or kidneys from small donors transplanted into larger recipients tend to fare worse, supporting a role for nephron “dosing” in transplantation.^153^–^158^

As with most clinical questions, a long duration of follow-up is necessary when looking for outcomes that may take many years to manifest. Giral et al. previously published a cohort of renal allograft recipients, with a mean of 32 months of follow-up, in whom they found no impact of graft weight on short-term graft survival.^159^ In their longer-term study, however, they used a donor kidney weight to recipient body weight (DKW/RBW) ratio of 2.3 to stratify recipients into two groups.^160^ The low DKW/RBW group showed a greater adaptive increase in GFR during the first 6 months post-transplant, which remained stable for 7 years but then declined faster after 7 years compared to the high DKW/RBW group. This observation suggests initial hyperfiltration in the smaller kidneys, which could not be sustained after 7 years, likely due to on-going nephron loss, as reflected in more proteinuria, more antihypertensive drug use, a greater degree of glomerulosclerosis, and a 55% increased risk for transplant failure by 2 years in the low DKW/RBW group. The authors conclude that incompatibility between graft and recipient weight is an independent predictor of long-term graft survival. These data strongly support the contention that nephron “dose”, relative to the recipient’s needs, should be an important consideration in organ allocation.

### Strategies for Optimization of Nephron Number

Evidence is emerging that clinically feasible interventions, at a critical period of nephron development, can rescue nephron number and impact later life blood pressure. In rats, adequate postnatal nutrition, achieved by cross-fostering growth-restricted pups onto normal lactating females at birth, was found to restore nephron number and abrogate development of subsequent hypertension.^73^ Similarly, supplementation of maternal low-protein diet with glycine, urea, or alanine during gestation normalized nephron number in all rat offspring, although blood pressure was only normalized in those supplemented with glycine.^71^ Postnatal hypernutrition in normal rats was found to increase nephron number by 20%, but these rats went on to develop hypertension and glomerulosclerosis with age, likely as a result of obesity.^72^ Vitamin A deficiency has been shown to reduce nephron number in a dose-dependent manner, but encouragingly a single dose of retinoic acid, administered during early nephrogenesis, was enough to restore nephron numbers to levels of control rats in pups exposed to a low-protein diet *in utero*.^30^,^161^ Interestingly, administration of ouabain was also found to abrogate the effect of serum starvation and low-protein diet on nephron development *in vitro* and *in vivo* again in rats.^162^ Although still preliminary, taken together, these studies suggest possible mechanisms whereby nephron numbers could be rescued if at-risk fetuses were identified early enough. Likewise, avoidance or judicious use of drugs during pregnancy, that are known to impact kidney development as described above, are another means to optimize fetal nephron number.^21^–^30^,^32^–^34^,^36^,^163^–^165^

## CONCLUSION

The idea that low nephron number may have a long-term impact on an individual’s later life risk of hypertension and renal disease has now entered the main stream. Until we learn more about developmental programming in nephrology, LBW should be used as the most useful current clinical surrogate for low nephron number and inborn risk of hypertension and renal disease. It is not surprising that nephron number and LBW are not the whole story, however. Other factors such as glomerular size, expression of sodium transporters, vascular reactivity, and high birth weight are also all important contributors and deserve more investigation. The demonstration that nephron numbers can be restored with timely intervention in experimental models points to plasticity within the system, making identification of individuals at risk and development of therapeutic tools even more urgent and compelling. Until such tools are developed, current evidence calls for optimization of perinatal care and early childhood nutrition as important strategies to help stem the growing epidemics of renal and cardiovascular disease in future generations.

## Abbreviations:

AGA: appropriate weight for gestational age;
BSA: body surface area;
CKD: chronic kidney disease;
DKW/RBW: donor kidney weight to recipient body weight;
ESRD: end-stage renal disease;
GDNF: glial cell-derived neurotrophic factor;
GFR: glomerular filtration rate;
HBW: high birth weight;
IUGR: intrauterine growth restriction;
LBW: low birth weight;
SGA: small for gestational age.
